# Antigen Stimulation Reactivates HIV-1 Proviruses Despite Integration in Repressive Chromatin

**DOI:** 10.64898/2026.06.11.731680

**Published:** 2026-06-12

**Authors:** Angelica Camilo-Contreras, Filippo Dragoni, Hao Zhang, Junlin Zhuo, Daniel Smith, Arturo Casadevall, Jun Lai, Pablo Tebas, Janet D. Siliciano, Robert F. Siliciano, Stuart Ray, Luis J. Montaner, Joel N. Blankson, Francesco R. Simonetti

## Abstract

Intact HIV-1 proviruses become progressively enriched in transcriptionally repressive genomic regions during long-term antiretroviral therapy (ART) and in elite controllers, raising questions about their capacity for reactivation in vivo. We used an antigen-restricted quantitative viral outgrowth assay (ag qVOA) to test whether cognate antigen stimulation can reverse latency of proviruses integrated within repressive chromatin. Using cells from two people with HIV (PWH) on ART, one on long-term treatment and one an elite controller, we show that antigen-specific stimulation induces viral outgrowth from intact proviruses integrated into a pericentromeric transition region and a zinc finger gene, respectively. These findings demonstrate that antigen recognition can overcome epigenetic constraints to reactivate proviruses with low inducibility and suggest that proviruses in so-called “deeper latency” may contribute to residual viremia and viral rebound following treatment interruption.

## Full Text Availability

The license terms selected by the author(s) for this preprint version do not permit archiving in PMC. The full text is available from the preprint server.

